# Reducing the Radioactive Surface Contamination Level of Cobalt-60-Contaminated Material with PVA-Glycerol-EDTA Combination Gel

**DOI:** 10.3390/gels11010056

**Published:** 2025-01-10

**Authors:** Rezky Anggakusuma, Gemilang Lara Utama, Muhammad Khoirul Zain, Kartini Megasari

**Affiliations:** 1 Doctoral Program on Environmental Sciences, Graduate School, Universitas Padjadjaran, Jl. Dipati Ukur No. 35, Bandung 40132, West Java, Indonesia; g.l.utama@unpad.ac.id; 2 Directorate of Laboratory Management, Research Facilities, and Science and Technology Park—BRIN, Jl. Sangkuriang No. 1–5, Bandung 40135, West Java, Indonesia; 3 Center for Environment and Sustainability Science, Universitas Padjadjaran, Jalan Sekeloa Selatan 1 No. 1, Bandung 40132, West Java, Indonesia; 4 Nuclear Chemical Engineering Study Program, Polytechnic Institute of Nuclear Technology—BRIN, Yogyakarta 55281, Central Java, Indonesia

**Keywords:** PVA, glycerol, EDTA, decontamination gel, Co-60, decontamination efficiency

## Abstract

Decommissioning of nuclear facilities can be performed in stages. One of the stages and processes in decontamination is the decontamination process before dismantling or facility area recovery activities. Decontamination can be performed using various methods, primarily physical and chemical. One chemical method involves using a gel made of polymers for decontamination. In this study, a gel consisting of a mixture of 15 g polyvinyl alcohol (PVA), 15 mL of glycerol, and 2 g Na-EDTA was dissolved in 100 mL. The three materials were dissolved in hot conditions until they dissolved, and a gel was formed. The formed gel was applied to the material contaminated by Co-60 with a radioactivity of 81 µCi, as much as 5 µL. The decontamination radioactive efficiency test results range from 53% to 98%, with the highest decontamination efficiency observed on glass media. This study also showed that higher EDTA concentrations can increase the ability of the PVA-glycerol gel to absorb and bind Co. This study also found that decontamination efficiency was influenced by the type of contaminated material and the concentration of EDTA. It can be concluded that gels with a composition of PVA, glycerol, and EDTA can reduce the level of contamination on the surface of materials made of glass, ceramics, and metal plates.

## 1. Introduction

The decommissioning of nuclear facilities has the potential for contamination, especially in areas that use open radioactive sources. If contamination occurs, radionuclide decontamination must be performed. The choice of decommissioning method also determines the type of decontamination to be chosen to reduce surface contamination in the dismantling process. The method or type of decontamination affects the overall cost of decommissioning. Decontamination costs not only consist of materials and consumables but also consider other costs such as labor costs and waste processing costs from decontamination [1,2]. Radionuclides can be moved on surfaces by physical mechanisms such as cutting or chopping, and chemical techniques can use foams, coatings, or ionic solutions [3].

For each decontamination method, the surface was sprayed with an acidic foam. Each layer was covered with polyvinyl acetate to reduce cross-contamination. The decontamination and covering of contaminated objects can reduce the level of radioactive waste from three to four [4]. During the decommissioning process of KKR 1 and 2 reactors (reactor TRIGA Mark II type), Korea decontaminated the buildings before they were destroyed and dismantled. Before and after demolition, a recheck was carried out to determine whether radionuclides remained. Suppose that the radionuclide identification results still show contamination. In this case, decontamination is conducted until the area or location reaches the permitted limits of applicable national and international laws [5].

Indonesia has the first research reactor and has the same type as KKR Korea, namely TRIGA 2000 Reactor Bandung, with an age of over 50 years and an operating permit that is about to expire [6,7]. Following applicable regulations, it is mandatory for the TRIGA 2000 Bandung reactor to prepare an appropriate decommissioning program with provisions of the Indonesian Nuclear Energy Regulatory Agency (BAPETEN) and International Atomic Energy Agency (IAEA) [8,9,10,11].

Based on experience in decommissioning research reactors, several radionuclides are contaminants in the research reactor area, including Co-60, Cs-134, Cs-127, Sr-90, U-238, I-129, I-131, Te-129, Ag-110, Th-232, Pu-238, Pu-239, Pu-240, Ir-129, Am241, Tc-97, Tc-98, Tc-99, Zr-93, Zr-95, Fe-55, Nb-94, and other radionuclides that are the result of fission reaction activation. In the IAEA technical reports of radionuclides often found in reactor decommissioning are included H-3, C-14, Na-22, Cl-36, Ar-39, Ca-41, Mn-54, Fe-55, Ni-59, Ni-63, Co-60, Zn-65, Mo-93, Zr-93, Nb-94, Ag-108 m, Ag-110 m, Sb-125, Ba-133, Cs-134, Eu-152, Eu-154, Eu-155, and Ho-166 m [12,13]. Decontamination is necessary before and after dismantling to remove contamination from the surface or area of the reactor facility. Several decontamination methods are available, including chemical, electrochemical, washing, and mechanical. The decontamination technique can be adjusted according to the contaminated place or media and type of radionuclide [12].

One decontamination technique uses a peel-off gel to decontaminate the surface of a material. He et al. conducted a study using gel or film materials, such as base polymers, as the decontamination method, which provided good decontamination results [14]. PVA is often used to prepare gels in various research fields, including research on PVA-based gels for decontamination of radioactive substances [15,16,17]. EDTA is often used to reduce contamination or waste caused by metal ions and hefty metals such as Cd, Pb, and Cs [18,19,20,21].

Owing to its non-toxicity, poly(vinyl alcohol) (PVA), a significant and adaptable non-ionic hydrophilic polymer, garnered much interest for its use as a hydrogel, among other applications. PVA gels are typically prepared using either chemical or physical techniques. PVA hydrogels that are chemically crosslinked can be produced multifunctionally [22,23]. Glycerol is a basic polyol with a prostereogenic center at position C_2_ and two primary and secondary hydroxyl groups. As a result, glycerol can undergo a wide range of chemical changes, including selective oxidation, dehydration, and hydrogenolysis, as well as selective protection and esterification, to provide a variety of valuable small-molecule building blocks with high chemical complexity for both polymer synthesis and small-molecule chemical synthesis [24].

In this study, a PVA-glycerol-EDTA gel was prepared to determine the effectiveness of surface decontamination of Co-60 radionuclides on plates or materials made of glass, metal, and ceramics. The experiment was conducted in a radioisotope laboratory, which is a part of the TRIGA 2000 BRIN Bandung reactor.

## 2. Results and Discussion

The first step in this study was to prepare a decontamination gel solution with varying PVA concentrations to determine the best gel composition to form a perfect and possibly peeled-off gel. Observation data for PVA decontamination gel formation can be obtained, as shown in Table 1.

**Figure 1:**
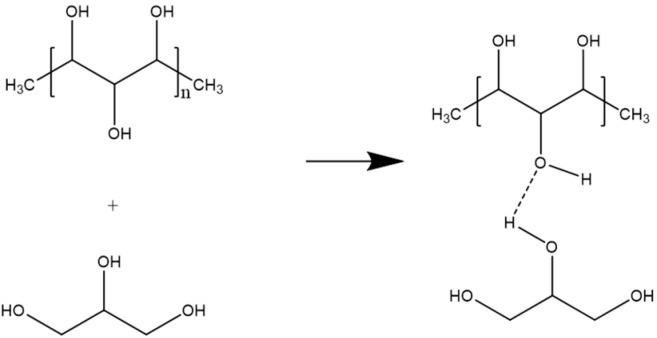
Crosslinking of PVA with glycerol.

From the data in Table 1, it can be concluded that the gel can ideally form, dry, and be peeled off with a PVA composition of 15% *w*/*v*. Mixing PVA and glycerol formed PVA-glycerol crosslinks. Increasing the PVA content can increase the possibility of crosslinking [25]. Therefore, a gel solution with a PVA concentration of 15% (*w*/*v*) was used in the next experiment. The PVA-glycerol crosslinks are shown in Figure 1.

When PVA is dissolved in the aqueous solution and mixed with glycerol, the chemical structure of PVA experiences changes, especially in the hydrogen bonds that dominate the PVA structure. Figure 1 shows random changes in the physical polymer chain due to the reaction of PVA and glycerol, causing crystalline dominance. The polymer can be explained by the presence of amorphous structures, which indicate that the polymer chains have begun to develop [26]. An appropriate composition of PVA and glycerol forms a perfect PVA-glycerol bond. A 15% *w*/*v* PVA gel solution was prepared for the decontamination test, using Na-EDTA as the chelating agent. The percentages of Na-EDTA added are shown in Table 2.

Adding EDTA as a chelating agent was expected to absorb more cobalt-60 to increase the effectiveness of decontamination. Hydroxyl radicals transfer reactivity from water to polymer chains by integrating macroradicals with H atoms. This reaction resulted in the formation of crosslinks. The crosslinking reaction is expressed as follows:

*PVA*(*H*) + *OH → PVA*
*H*_2_*O*

2PVA* glycerol → *PVA-glycerol-PVA(Crosslinked Network)*

*EDTA(H)* + *OH* → EDTA* + *H*_2_*O*

PVA-glycerol-PVA* + EDTA* → *PVA-glycerol-PVA-EDTA*

*PVA-glycerol-PVA-EDTA* + *Co-60* → *Co60-PVA-glycerol-PVA-EDTA-Co-60*.

EDTA may be the key to the cobalt-60 decontamination mechanism found in this study. Research conducted by Mudsainiyan et al. states that EDTA is the most desirable ligand because of several conditions and a flexible connection mode. This ligand has four carboxylate groups with potentially ten coordination arms (eight O atoms and two N atoms) [27].

### 2.1. Cobalt-60 Decontamination Effectiveness Testing

The PVA-EDTA synthetic solution was tested on test media in ceramic, glass, and metal plates dripped with 5 µL of cobalt-60 radioactivity of 81 µCi. Count measurements were performed using a Nuvia brand Contamination Monitor tool with the CoMo 170 type. Tests for each medium in each solution were performed in triplicate. Data on the effectiveness of each solution and the contamination medium are presented in Table 3.

This study achieved the highest decontamination using 15% *w*/*v* PVA gel with 2% *w*/*v* EDTA, with an effectiveness value above 90 percent. The effectiveness of gel decontamination on metal media is high on smooth media such as Teflon or glass, which is consistent with the results of research conducted by Gurau and Deju (2015), who used the commercial decontamination gel DeconGel™ 1101, with the decontamination effectiveness value for cobalt-60 being above 90% [28]. Combining physical and chemical methods can achieve decontamination effectiveness values > 90% in all media [3].

The presence of EDTA, which can bind cobalt ions and adhere to the surface layer of the material, can increase the effectiveness of the decontamination. The EDTA levels can affect the ability of the gel to bind to cobalt ions. Low EDTA levels (<2%) resulted in no binding between EDTA and cobalt ions because the EDTA in the gel was already saturated with PVA and glycerol. Meanwhile, high EDTA levels (>2%) cause saturation and EDTA cannot dissolve in PVA-glycerol. Dissolution can be seen from the white parts or EDTA powder, which does not dissolve when the gel dries.

The effectiveness of decontamination on glass is the highest, owing to its smooth surface. The smooth surface of the glass causes the attractive force between the glass and the cobalt ions to be smaller so that the cobalt solution does not enter the pores or gaps on the surface of the glass layer. The surfaces of the ceramics and metal plates have a rougher texture and tiny pores or gaps. Small pores or gaps can cause the Co solution to enter, making it difficult to interact with the decontamination gel. Therefore, further studies on surfaces with rough textures and pores are required to determine the appropriate gel concentration for achieving a higher bonding capacity [29]. PVA gel has good porosity, so it can be used as an absorbent material for heavy metals. This was found in research conducted by Wang (2016); Wang studied the use of composite hydrogels to remove or reduce heavy metal contamination [15].

From Table 3, a statistical analysis was conducted to determine whether there was a difference between each decontaminated material. The study results show that the *p*-value for the one-sample t-test of the ceramic, glass, and metal plate materials was <0.001. This value was less than 0.05, indicating that the decontamination of the same material with different concentrations was significantly different. This means that different concentrations of EDTA on the same material will provide different decontamination effects. Meanwhile, for the ANOVA test to determine whether there was an effect between the material and the concentration of EDTA, the *p*-value was 0.001, which was less than 0.05. It can be concluded that, in this study, the concentration of EDTA and the type of material greatly influenced the efficiency of Co-60 radioactivity decontamination.

With high decontamination effectiveness, previously contaminated materials can be classified as goods or waste with a lower category, and if it is at a safe level, the material can be classified as ordinary waste or domestic waste. The gel used for decontamination has been contaminated with radioactive substances into an active film. Film waste will have less volume and weight. Active film can be processed as active waste according to the type of radionuclide. Appropriate waste management will reduce the amount of radioactive waste and the cost of waste management.

### 2.2. FTIR Analysis

**Figure 2:**
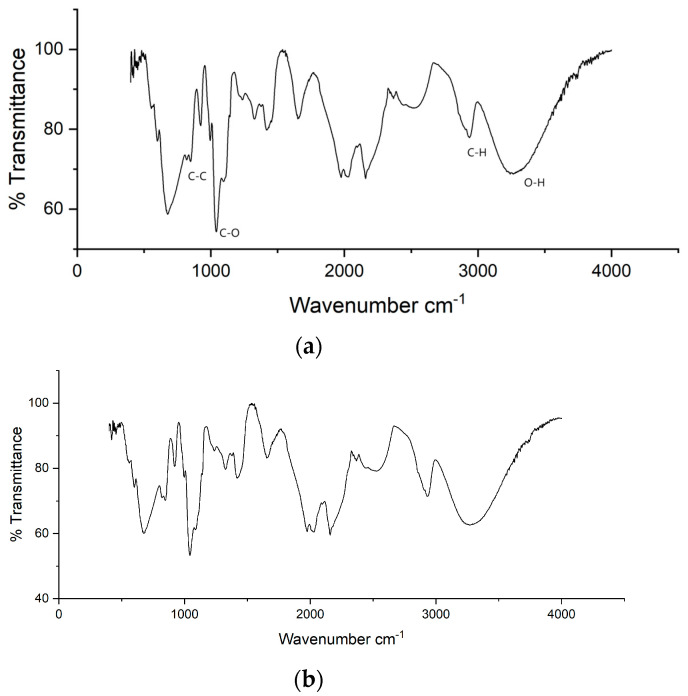
Result of FTIR analysis. (**a**) Spectrum of PVA-glycerol; (**b**) Spektrum PVA-glycerol-Co 60.

**Figure 3:**
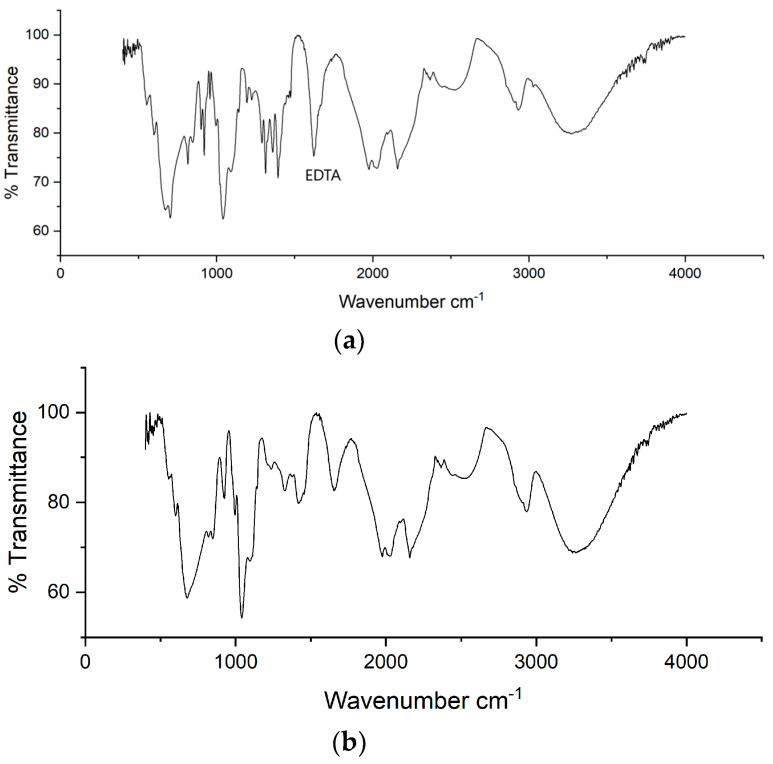
Result of FTIR analysis. (**a**) Spectrum of PVA-glycerol-EDTA; (**b**) Spektrum PVA-glycerol-EDTA-Co 60.

Figure 2 and Figure 3 show a peak at a wavelength of 3100–3800 nm, which indicates the presence of an O-H group or non-hydrogen bond. For a specific spectrum for glycerol there is a wavelength 849 cm^−1^ C-C stretching; 1030 cm^−1^ C-O stretching; 1416 cm^−1^, 908 cm^−1^ CH_2_; 1108 cm^−1^ C-OH; 2880 and 2932 cm^−1^ C-H stretching; and 3286 cm^−1^ O-H stretching [30]. For PVA, approximately 2500–3500 corresponds to the bonded hydroxyl groups. The peak observed at 2946 cm^−1^ can be assigned to the C-H group [31].

It is known that there are prominent peaks at wavenumbers 1600 cm^−1^–1700 cm^−1^ and 2900 cm^−1^. This peak is typical of EDTA, indicating the presence of a carboxylate group in the compound. EDTA ligand compounds, both those containing metal and those free of complexing metals, have infrared absorption peaks that are generally similar. However, if there are different complexing metals, there is usually a shift in the typical absorption peak for each metal atom [32]. The reaction between the compound of cobalt metal ions and EDTA ligands results from the coordination between EDTA ligands and cobalt ions. Cobalt ions are bound by coordination with the results of electron acquisition from oxygen atoms originating from the hydroxide groups. This can occur because oxygen atoms have free electron pairs that can be donated to cobalt ions. The characteristic peak absorption at 1040 cm^−1^ comes from the Co-O stretching vibration. The absorption peak at 1250 cm^−1^ is thought to originate from the Co-N stretching vibration due to a coordinate covalent bond in the compound [Co(EDTA)]^−^. A coordinate covalent bond is a covalent bond that occurs because of the sharing of a pair of electrons that unites one atom. In this case, the empty orbitals in cobalt-60 can form a complex with the entry of free electrons from EDTA. An absorption peak at 2140 cm^−1^ indicates the presence of group coordination in the C=C=O bond with cobalt [33].

### 2.3. SEM Analysis

**Figure 4:**
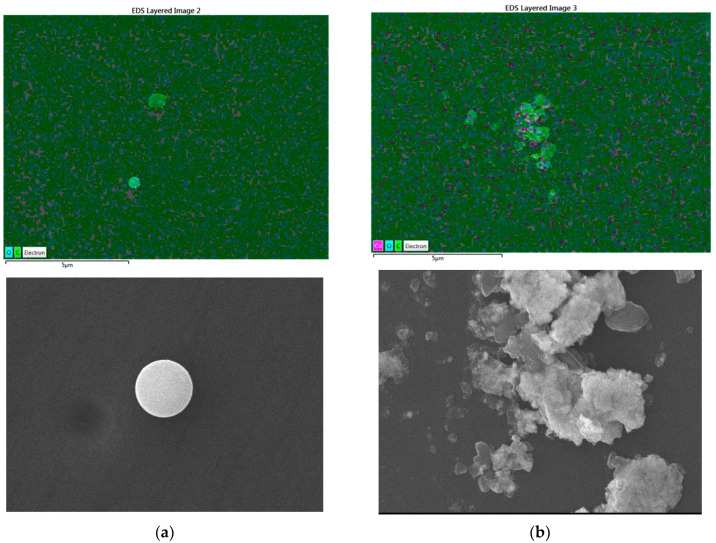

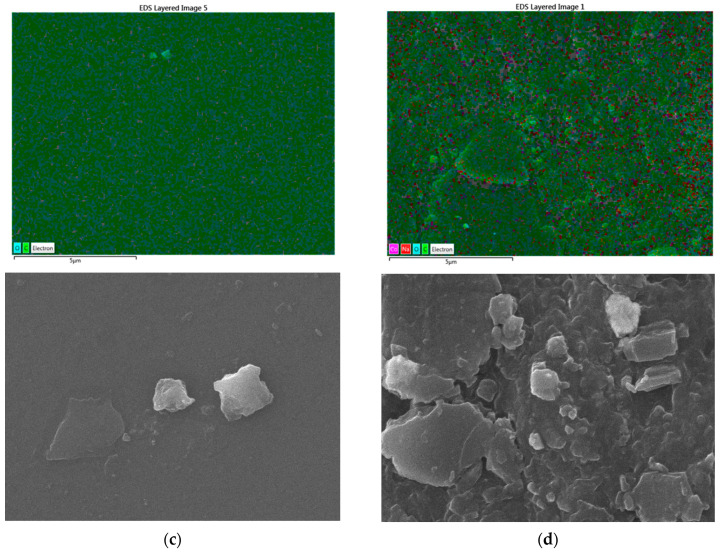
Result from SEM EDX analysis.with a magnification of 5000 times (**a**) PVA-glycerol; (**b**) PVA-glycerol-Co; (**c**) PVA-glycerol-NaEDTA; and (**d**) PVA-glycerol-NaEDTA-Co.

The SEM-EDX results show a significant difference between the PVA film with EDTA and the PVA-EDTA gel that interacts with Co. A significant difference show at Figure 4 The PVA films that were in contact with Co exhibited a denser structure. The results of component identification also show the presence of Co, which originated from contamination. From the results of this experiment, it can be concluded that PVA, with or without EDTA, can interact with and bind Co.

### 2.4. XRD Analysis

**Figure 5:**
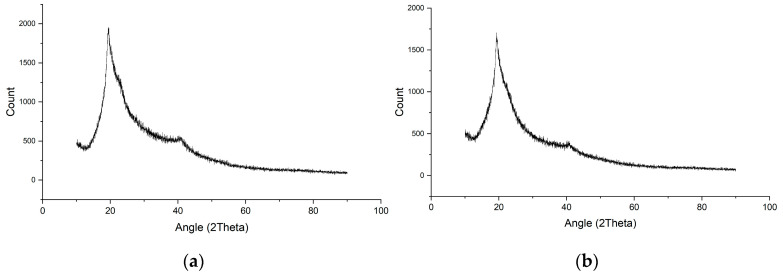

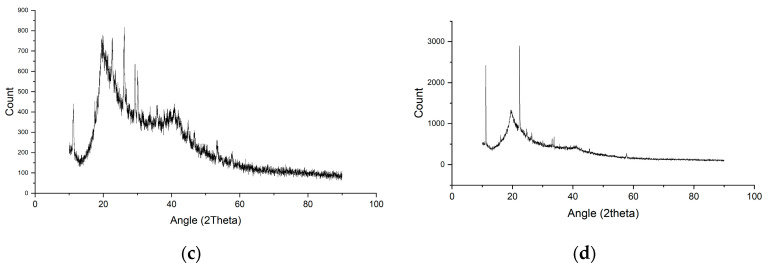
XRD measurement result. (**a**) PVA-glycerol; (**b**) PVA-glycerol-Co; (**c**) PVA-glycerol-NaEDTA; and (**d**) PVA-glycerol-NaEDTA-Co.

The XRD analysis results show at Figure 5 that all PVA-glycerol-metal film samples have an amorphous percentage above 65%, indicating that no chemical interactions can form crystals. Amorphous polymers have irregular crystalline structures. Amorphous polymers are flexible and transparent. The film resulting from the reaction between the PVA-glycerol gel and metal ions is included in the semi-crystalline amorphous group because there are still less than 35% crystals [34]. Crystallites in the film can come from the metal ions Na-EDTA and Co-EDTA, which do not fully react, interact, and bond with the O-H groups originating from the polymer gel.

### 2.5. XRF Analysis

Table 4 presents the analysis results of the Co concentration lifted and attached to the film.

Nonetheless, the Co ions in the film used in our investigation had a greater concentration of metal ions. One possible explanation for the rise in metal ion concentration, as determined by XRF, is erosion brought on by water evaporation, which raises the level above the concentration [35,36,37]. However, the contact or absorption of metal ion solutions between the pores of the media may decrease concentration. Based on the data in Table 4, it can be concluded that the addition of EDTA affected the binding of Co. This can be observed from the increase in the concentration of Co in the film. Further research is necessary to review the effects of contaminant evaporation and contact time. In addition, a study of the impact of contact time, contact temperature, and the concentration of contaminants that can be decontaminated is also needed.

## 3. Conclusions

In this study, gel synthesis was performed using a combination of PVA, glycerol, and EDTA. The combination that can be formed perfectly and dried into a film at 25–27 °C for 24 h is PVA with a concentration of 15% *w*/*v* and an additional EDTA of 2% *w*/*v*. From the results of observations of the gel composition, the amount of PVA < 15% *w*/*v* could not form the film because the gel did not dry perfectly. If PVA > 15% *w*/*v*, it forms lumps because of the saturation of the solution, and PVA does not dissolve completely. The structure of the PVA-glycerol-EDTA gel functional group can be seen from the results of the FTIR test; the typical peaks of each component are apparent, and crosslinking between PVA and glycerol is also evident. The addition of EDTA > 2% *w*/*v* resulted in the crystallization of the gel solution, and the film formed was not transparent and stiffer. Adding EDTA can also increase Co’s absorption or binding, thereby increasing Co’s levels during decontamination. Cobalt reacts with the nanoparticles of the PVA-glycerol-EDTA gel via electrostatic interactions. Metal ions interact with PVA-glycerol’s hydroxyl groups (O-H). PVA-glycerol gel has many hydroxyl groups because the crosslinking between PVA and glycerol forms a polymer. The O-H and N groups in EDTA can increase the binding of Co; this can be seen in the XRF test results, which show an increase in concentration in gels with higher EDTA concentrations. The film resulting from the reaction between PVA-glycerol-EDTA and Co formed an amorphous material.

## 4. Material and Methods

### 4.1. Tools and Materials

**Figure 6:**
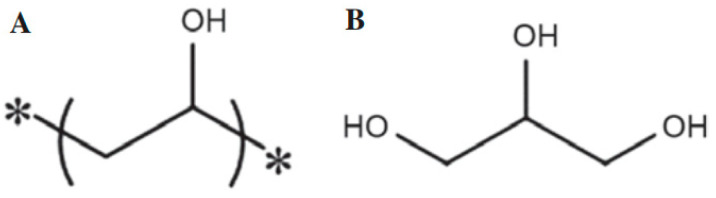
The chemical structure of (**A**) polyvinyl alcohol (PVA) * is many polymers and (**B**) glycerol [38].

The tools used in this study included measuring cups, beakers, spatulas, hot plates, micropipettes, contamination monitors (Nuvia CoMo-170, NUVIATech Instruments, Rueil-Malmaison, France), glass, ceramic, metal media, mica tube molds (d = 3 cm; h = 0.5), zip plastic, tweezers, and glue guns. The materials used included a Co-60 solution with radioactivity of 81 µCi, polyvinyl alcohol (PVA), distilled water, glycerol 98%, and Na-ethylene diamine tetraacetate (EDTA). The chemical structures of PVA and glycerol are shown in Figure 6.

### 4.2. Making PVA Gel

The PVA gel is made from a mixture of PVA, glycerol, distilled water, and ethanol. PVA with specific weights (5 g (PVA 5%), 10 g (PVA 10%), 15 g (15%), and 20 g (20%)) was dissolved in 50 mL of distilled water and then heated at 90 °C until the PVA dissolved. After the PVA was dissolved, 9 mL of glycerol was added to a volume of 100 mL. All the ingredients were stirred until they were completely dissolved. After all the ingredients were mixed, a white gel formed, which became apparent after standing for 24 h at room temperature (26–27 °C). The PVA-EDTA gel was prepared by adding EDTA with specific weights (1 g (1%), 2 g (2%), 3 g (3%), and 5 g (5%).

### 4.3. Contaminated Material Creation

**Figure 7:**
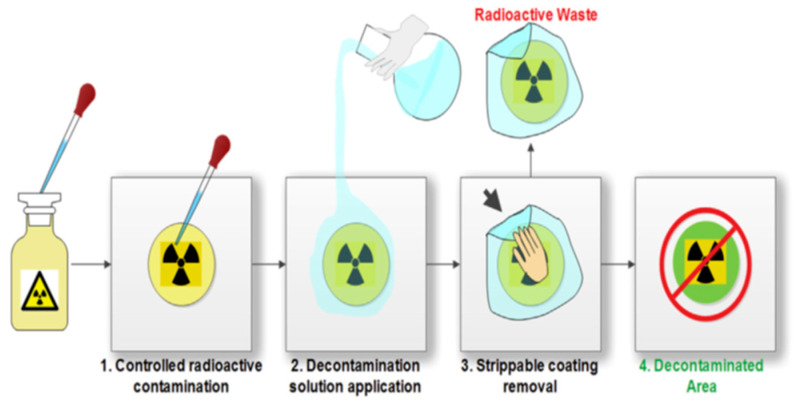
Radiation decontamination procedure using PVA solution [39]. A green mark is an area after decontamination with gel PVA-glycerol-EDTA and indicates decreased level contamination Co-60.

Glass, ceramic, and steel plate media were installed in a mold made from plastic mica that measures 3 cm in diameter. In the middle section, print a drip of five microliters of Co-60, a radioactivity of 81 µCi solution (for efficiency decontamination test), and then wait until dry. For XRF analysis, we used Co with 10,000 ppm concentration. The spot where Co, the level of contamination, was detected was monitored directly at a distance of 1 cm from the surface. PVA or PVA-EDTA gel was added until the cover was printed. Preparation was conducted for storage in a temperature room (25–27 °C for 24 h). Illustration processing can be seen in Figure 7.

### 4.4. Decontamination Effectiveness Calculation

After 24 h, the gel was peeled off from the surface. Then, the gel and surface contamination were measured to return the level of contamination on the surface. The effectiveness of the decontamination was calculated using the following formula:

$$K\left(\%\right)=\left[\frac{Ai-Af}{Ai}\right]\times 100$$

with:

K = effectiveness of decontamination in percent,

Ai = initial media activity in cps units,

Af = media activity after the peeling gel was removed in cps units.

Statistical analysis was used to determine whether there was a difference and influence on the test variables, such as EDTA levels and types of decontaminated materials. The statistical analyses used were a one-sample *t*-test and ANOVA.

### 4.5. Characterization of Material

1. Fourier transform infrared (FTIR) spectroscopy identifies and analyszes the substance’s chemical makeup and structure. The instrument used was an ATR Diamond Thermo Nicolet iS5, Thermo Fisher Scientific Inc., Madison, WI, USA. Wavenumber 400–4000 cm^−1^.
2. Scanning electron microscopy (SEM) is used to study the surface morphology, composition, and structure of the materials. The instruments used were the JEOL JSM-IT300 + OXFORD EDS 3-1-2 Musashino, Akishima, Tokyo, Japan, for magnification until 5000.
3. X-ray diffraction (XRD) was used to study the crystallographic structures, phase compositions, and physical properties of the materials. The instruments used were the Bruker AXS D8 Eco Bruker Corporation 40 Manning Road Billerica, MA, USA. With 2 Theta 10° to 90°.
4. X-ray fluorescence (XRF) was used to determine the elemental composition of the materials. The instruments used were the Thermo Scientific/Niton XL3t, Thermo Fisher Scientific Inc., Madison, WI, USA.

## Figures and Tables

**Table 1:** Observation results of gel formation tests with PVA variations.

Solution	Synthesis Process	Gel Formation	Dry Gel	Gel Peel	Final Observation Results
Solution A (5% *w*/*v* PVA)	Yes	No	No	No	The gel does not form
Solution B (10% *w*/*v* PVA)	Yes	Yes	No	No	Gel forms but is wet and cannot be peeled off
Solution C (15% *w*/*v* PVA)	Yes	Yes	Yes	Yes	It forms a gel, dries perfectly, and can be peeled off.
Solution D (20% *w*/*v* PVA)	Yes	Yes	Yes	Yes	It forms a gel, dries perfectly, and can be peeled off.

**Table 2:** Na-EDTA composition in each solution variation adding to PVA 15% *w*/*v*.

No.	Solution Code	% Na-EDTA
1	Gel 1	0% *w*/*v*
2	Gel 2	1% *w*/*v*
3	Gel 3	2% *w*/*v*
4	Gel 4	3% *w*/*v*
5	Gel 5	5% *w*/*v*

**Table 3:** Rate effectiveness of EDTA variations of PVA gel decontamination on glass, ceramic, and metal plate media.

No.	Contamination Media	Average Decontamination Effectiveness (%)				
		Gel 1	Gel 2	Gel 3	Gel 4	Gel 5
1.	Ceramics	82	88	95	78	53
2.	Glass	95	93	98	80	89
3.	Metal Plate	67	94	95	54	75

**Table 4:** Concentration Co at film.

Film	Co (10.000 ppm)
PVA-glycerol	<LOD *
PVA-glycerol-EDTA	<LOD *
PVA-glycerol-Co	35.166 ppm
PVA-glycerol-EDTA-Co	82.923 ppm

* LOD Co = 25 ppm.

## Data Availability

The original contributions presented in this study are included in the article. Further inquiries can be directed to the corresponding author.
